# The composition and stability of the vaginal microbiota of normal pregnant women is different from that of non-pregnant women

**DOI:** 10.1186/2049-2618-2-4

**Published:** 2014-02-03

**Authors:** Roberto Romero, Sonia S Hassan, Pawel Gajer, Adi L Tarca, Douglas W Fadrosh, Lorraine Nikita, Marisa Galuppi, Ronald F Lamont, Piya Chaemsaithong, Jezid Miranda, Tinnakorn Chaiworapongsa, Jacques Ravel

**Affiliations:** 1 Perinatology Research Branch, Program for Perinatal Research and Obstetrics, Division of Intramural Research, Eunice Kennedy Shriver National Institute of Child Health and Human Development, NIH, Bethesda, MD and, Detroit, MI, USA; 2 Department of Obstetrics and Gynecology, University of Michigan, Ann Arbor, MI, USA; 3 Department of Epidemiology and Biostatistics, Michigan State University, East Lansing, MI, USA; 4 Department of Obstetrics and Gynecology, Wayne State University School of Medicine, Detroit, MI, USA; 5 Institute for Genome Sciences, University of Maryland School of Medicine, Baltimore, MD, USA; 6 Department of Microbiology and Immunology, University of Maryland School of Medicine, Baltimore, MD, USA; 7 Department of Obstetrics and Gynaecology, University of Southern Denmark, Odense, Denmark; 8 Division of Surgery, University College, Northwick Park Institute for Medical Research Campus, London, UK

**Keywords:** Community stability, Longitudinal sampling, Pregnancy, Vaginal microbiome, *Lactobacillus*, Dynamics

## Abstract

**Background:**

This study was undertaken to characterize the vaginal microbiota throughout normal human pregnancy using sequence-based techniques. We compared the vaginal microbial composition of non-pregnant patients with a group of pregnant women who delivered at term.

**Results:**

A retrospective case–control longitudinal study was designed and included non-pregnant women (n = 32) and pregnant women who delivered at term (38 to 42 weeks) without complications (n = 22). Serial samples of vaginal fluid were collected from both non-pregnant and pregnant patients. A 16S rRNA gene sequence-based survey was conducted using pyrosequencing to characterize the structure and stability of the vaginal microbiota. Linear mixed effects models and generalized estimating equations were used to identify the phylotypes whose relative abundance was different between the two study groups. The vaginal microbiota of normal pregnant women was different from that of non-pregnant women (higher abundance of *Lactobacillus vaginalis*, *L. crispatus*, *L. gasseri* and *L. jensenii* and lower abundance of 22 other phylotypes in pregnant women). Bacterial community state type (CST) IV-B or CST IV-A characterized by high relative abundance of species of genus *Atopobium* as well as the presence of *Prevotella*, *Sneathia*, *Gardnerella*, *Ruminococcaceae*, *Parvimonas*, *Mobiluncus* and other taxa previously shown to be associated with bacterial vaginosis were less frequent in normal pregnancy. The stability of the vaginal microbiota of pregnant women was higher than that of non-pregnant women; however, during normal pregnancy, bacterial communities shift almost exclusively from one CST dominated by *Lactobacillus* spp. to another CST dominated by *Lactobacillus* spp.

**Conclusion:**

We report the first longitudinal study of the vaginal microbiota in normal pregnancy. Differences in the composition and stability of the microbial community between pregnant and non-pregnant women were observed. *Lactobacillus* spp. were the predominant members of the microbial community in normal pregnancy. These results can serve as the basis to study the relationship between the vaginal microbiome and adverse pregnancy outcomes.

## Background

The human vagina and the bacterial communities that reside therein represent a finely balanced mutualistic association [1]. Since the report (and discovery) of *Lactobacillus* (Döderlein Bacillus) as common inhabitants of the human vagina in 1892 by Gustav Döderlein, it is common wisdom that *Lactobacillus* is a keystone genus in the vagina [2-4]. The presence of *Lactobacillus* spp. is associated with a healthy state and is thought to protect reproductive age women from non-indigenous pathogens [5-26], certainly by contributing to the maintenance of a low vaginal pH (<4.5) through the production of lactic acid [24,27-34]. The vaginal microbiota is unique as it undergoes major compositional changes throughout a women’s lifespan from birth, to puberty and menopause [35-41]. Very little is known about the composition of the vaginal microbiota throughout these transitional stages, but it appears that sex steroid hormones play major roles in driving the composition and stability of the vaginal microbiota [39,42-49].

The development of culture-independent profiling methods to detect fastidious or non-cultivable organisms through the analysis of the sequence of marker genes, such as the 16S rRNA gene, has precipitated a revolution in biology and medicine, by spurring projects such as the National Institutes of Health (NIH)-funded Human Microbiome Project [50-56], the European MetaHit project [57] and the creation of the International Human Microbiome Consortium. Culture-based analyses have been used for decades and have contributed critical knowledge about the microbes inhabiting the human body, including the vagina, and the understanding of infectious diseases that affect the genital tract [17,58-71]. However, cultivation techniques are laborious, time-consuming, and quantitative microbiology of polymicrobial infection or complex ecosystems is challenging when trying to accurately assess the contribution of each organism to the microbial population structure [72-74]. Moreover, many organisms cannot be cultured because the essential requirements for growth are not known [72,75,76]. Advances in cultivation techniques continue to occur and are sometimes informed by the results of sequence-based methods [73,77-79].

Culture-independent characterization of bacterial communities can be generated using the amplification and sequencing of the 16S rRNA gene [80-83] or metagenomics approaches in which the sequences of the bacterial community genes and genomes are obtained [51,57,76-78,84-88]. However, 16S rRNA gene profiling is widespread and has been used for the discovery of important clinically relevant organisms which had resisted cultivation for decades [76,77,89-91]. This method is also affordable and rapid, and results are tractable from an analytical point of view. The use of molecular culture-independent techniques has increased the knowledge about the complexity of the microbial ecosystem of multiple body sites, including the human vagina [21,26,40,41,47,76,92-110].

Most of the data published to date on the human vagina microbial ecosystem focused on healthy asymptomatic non-pregnant women of reproductive age [100,109,111,112]. These studies have established that at least six types of vaginal microbiota exist, referred to as community state types (CSTs) [100,109,112,113]. Four of these CSTs are most often dominated by one of four *Lactobacillus* spp. commonly found in the vagina (*L. crispatus*, *L. iners*, *L. jensenii* and *L. gasseri*), while the remaining two lack substantial numbers of *Lactobacillus* spp. and are composed of a diverse array of anaerobic bacteria including species associated with bacterial vaginosis such as *Prevotella*, *Megasphaera*, *Gardnerella vaginalis*, *Sneathia* and *Atopobium vaginae*[13,96,102,105,114-122]. While these two states are found in otherwise healthy asymptomatic women, they are often associated with high Nugent scores [123], a Gram stain method used in the diagnosis of bacterial vaginosis in research settings [61,71,124-126]. High Nugent scores or changes in the vaginal microbiota have been associated with increased risk of sexually transmitted infections [20,127-138], including HIV [10,14,22,99,139-150], preterm birth [62,108,151-203], and adverse perinatal outcomes such as post-abortal sepsis [204], early and late miscarriage [165,205,206], recurrent abortion [205], histological chorioamnionitis [160,164] and postpartum endometritis [183,207].

Interestingly, in some women the vaginal microbiota is remarkably dynamic (it can change over a short period of time from *Lactobacillus* dominated CSTs to CSTs lacking a substantial number of *Lactobacillus* spp.), while in other women it is relatively stable [100,112]. Menstruation and sexual activity have been shown to have negative effects on the stability of the vaginal microbiota [26,42,43,112,208-210]. The secretory phase of the menstrual cycle, which is characterized by high concentrations of estrogen and progesterone, appears to be more stable in terms of microbial community composition [112].

Knowledge of the vaginal microbiota throughout pregnancy is sparse, and only a few studies have examined the vaginal microbiota in pregnant women using culture-independent methods [211-213], and none analyzed samples collected longitudinally throughout pregnancy from the same women using 16S rRNA gene sequence-based methods. Using a Gram stain scoring system, cultivation and terminal restriction fragment length polymorphism, Verstraelen et al. demonstrated the importance of *L. crispatus* and *L. gasseri* in maintaining stability in a population of Dutch women sampled once in each trimester [211]. The consensus from previous studies is that *Lactobacillus* spp. predominate the vaginal microbiota during pregnancy; this observation is consistent with the results of a recent 16S rRNA gene sequence-based cross-sectional study reported by Aagaard and colleagues [213]. None of these studies examined the degree of stability in the vaginal microbiota during pregnancy using 16S rRNA gene sequence analysis. Stability and resilience of ecosystems are now recognized to be important in understanding the fitness of the community, as well as the response to perturbations [56,214-220]. Therefore, studies of the microbiota in several body sites are characterizing stability and resilience, as well as how they relate to health and disease [221-233].

The purpose of this study was to characterize the changes in the composition of the vaginal microbiota of pregnant women followed longitudinally (over the duration of pregnancy). The control group consisted of non-pregnant women who were frequently sampled. Here we report the use of 16S rRNA gene sequence-based methods to characterize the vaginal microbiota of normal pregnant women and the differences observed between these and non-pregnant subjects. The two major findings were that the microbial composition of the vaginal microbiota in normal pregnancy is different from that of non-pregnant women; moreover we demonstrate, for the first time, that the vaginal microbiota during pregnancy is more stable than in the non-pregnant state.

## Methods

### Study design

This was a prospective longitudinal cohort study to characterize changes in the vaginal microbiota in normal pregnant and non-pregnant women. A normal pregnancy was defined as a woman with no obstetrical, medical or surgical complications, who agreed to participate in this study, provided written signed informed consent, and delivered at term (38 to 42 weeks) without complications. Non-pregnant women were of reproductive age and free of clinical disease [112]. These patients were enrolled in a prospective study designed to describe the vaginal microbiota as a function of time. Details of this study have been previously reported [112].

### Study procedures

Pregnant women who agreed to participate in the longitudinal study had a speculum examination at each visit and a sample of vaginal fluid was collected under direct visualization from the posterior vaginal fornix by an obstetrician or a midwife using a Dacron swab (Medical Packaging Swab-Pak™, Camarillo, CA, USA). Samples were collected every 4 weeks until 24 weeks of gestation, and every 2 weeks until the last prenatal visit. Samples were stored at -70°C until assayed. Non-pregnant patients were self-collected sampled twice weekly for 16 weeks using validated methods previously described [112,234]. All samples were Gram-stained and analyzed using the Nugent score [61]. The use of samples from the longitudinal study of pregnant women was approved by the Human Investigations Committee of Wayne State University and the Institutional Review Board of the *Eunice Kennedy Shriver* National Institute of Child Health and Human Development. The data from non-pregnant women are derived from a previous study [112] and are publicly available in the sequence read archive (accession no. SRA026073). The metadata associated with the sequence data are available in dbGap (dbGap study no. phs000261).

### DNA extraction, amplification and pyrosequencing of barcoded 16S rRNA genes

Genomic DNA was extracted from archived vaginal swab specimens. Procedures for the extraction of genomic DNA from frozen vaginal swabs have been developed, validated and previously published [109]. Briefly, frozen vaginal swabs were immersed in 1 ml pre-warmed (55°C) cell lysis buffer, composed of 0.05 M potassium phosphate buffer containing 50 μl lyzosyme (10 mg/ml), 6 μl mutanolysin (25,000 U/ml; Sigma-Aldrich, St. Louis, MO, USA) and 3 μl lysostaphin (4,000 U/ml in sodium acetate; Sigma-Aldrich). The mixture was incubated for 1 hour at 37°C followed by the addition of 10 μl proteinase K (20 mg/ml), 100 μl 10% SDS, and 20 μl RNase A (20 mg/ml), and the mixture was incubated for 1 hour at 55°C. The samples were then transferred to a FastPrep Lysing Matrix B tube (MP Biomedicals, Santa Ana, CA, USA) and microbial cells were lysed by mechanical disruption using a bead beater (FastPrep instrument, MP Biomedicals) set at 6.0 m/s for 30 seconds. The lysate was processed using the ZR Fecal DNA extraction kit (ZYMO Research, Irvine, CA, USA) according to the manufacturer’s recommendation and omitting the lysis steps (steps 1 to 3). The kit included a column (Zymo-Sin IV-HRC spin filter) specifically designed to remove PCR inhibitors from DNA samples. The DNA was eluted into 100 μl TE buffer, pH 8.0. This procedure provided between 2.5 and 5 μg of high quality whole genomic DNA from vaginal swabs.

Universal primers 27F (Forward) and 338R (Reverse) were used for PCR amplification of the V1-V2 hypervariable regions of 16S rRNA genes [112]. The 338R primer included a unique sequence tag to barcode each sample. The primers were as follows: 27F-5′-*GCCTTGCCAGCCCGCT*CAGTC**AGAGTTTGATCCTGGCTCAG**-3′ and 338R-5′-GCCTC *CCTCGCGCCATCAG*NNNNNNNN**CATGCTGCCTCCCGTAGGAGT**-3′, where the italicized sequences are the 454 Life Sciences FLX sequencing primers B and A in 27 F and 338R, respectively, and the bold font denotes the universal 16S rRNA gene primers 27 F and 338R. The 8-bp barcode within primer 338R is denoted by eight Ns. Using 96 barcoded 338R primers [109], the V1-V2 regions of 16S rRNA genes were amplified in 96 well microtiter plates using AmpliTaq Gold DNA polymerase (Applied Biosystems, NY, USA) and 50 ng template DNA in a total reaction volume of 50 μl. Reactions were run in a PTC-100 thermal controller (BioRad, Hercules, CA, USA) using the following cycling parameters: 5 minutes denaturation at 95°C, followed by 20 cycles of 30 seconds at 95°C (denaturing), 30 seconds at 56°C (annealing) and 90 seconds at 72°C (elongation), with a final extension at 72°C for 7 minutes. Negative controls without a template were included for each barcoded primer pair. The presence of amplicons was confirmed by gel electrophoresis on a 2% agarose gel and staining with SYBRGreen (Life Technologies, Carlsbad, CA, USA). PCR products were quantified using the Quant-iT™ PicoGreen® dsDNA assay (Life Technologies). Equimolar amounts (100 ng) of the PCR amplicons were mixed in a single tube. Amplification primers and reaction buffer were removed from each sample using the Agencourt AMPure Kit (Beckman-Coulter, Pasadena, CA, USA). The purified amplicon mixtures were sequenced by 454 FLX Titanium pyrosequencing using 454 Life Sciences® primer A by the Genomics Resource Center at the Institute for Genome Sciences, University of Maryland School of Medicine using protocols recommended by the manufacturer as amended by the Center and previously described [109].

### Sequence analysis

Sequences were binned by samples using the sample-specific barcode sequences and trimmed by removal of the barcode and primer sequences. Sequence read quality check was performed using a bioinformatics pipeline that is in accordance with NIH Human Microbiome Project (HMP) standard operating procedures [109]. Briefly, raw sequence reads were filtered to meet the following criteria: 1) minimum and maximum read length of 200 bp and 400 bp; 2) no ambiguous base calls; 3) no homopolymeric runs longer than 8 bp; 4) a read was discarded if the average quality value was less than q25 within a sliding window of 50 bp; 5) a read was discarded if it was identified as a putative chimeric sequence by UCHIME [235]. The sequences that passed the above filtering procedure were denoised in order to correct for potential sequencing errors at 99% level using UCLUST [235]. Sequences were then taxonomically classified using pplacer version v1.1.alpha08 [236]. pplacer makes taxonomic assignment using a linear time maximum-likelihood method (or alternatively a Bayesian phylogenetic placement method) using a community specific reference tree. Version 0.2 of the vaginal community 16S rRNA gene reference tree was employed. Overall, 86% of all sequence reads that passed quality control criteria in this study were classified to the species level, and 57% of the reads were taxonomically assigned to the genus *Lactobacillus*.

### Statistical analysis

In microbiology, the abundance of bacteria is measured in a logarithmic scale (base 10), given the wide range of bacterial abundance and the exponential nature of bacterial growth under certain circumstances (for example, *in vitro*). Therefore, it is the norm to compare microbial abundance over time using the difference of logs, log_10_ (p) - log_10_ (q), which is the same as the log fold change log_10_ (p/q), where p and q are relative abundances of a given microorganism in two samples.

Estimating changes in abundance of a complex microbial ecosystem within a patient at two time points becomes more challenging, as several microorganism types (phylotypes) need to be considered. In order to address this challenge, we assessed the dissimilarity between two community states (in other words, how divergent two community states are) using the Jensen-Shannon metric [237]. The term “community state” in microbial ecology refers to the relative abundance of all phylotypes at a particular time point in a subject; in our case, a sample of vaginal fluid.

The Jensen-Shannon divergence between two community states, p and q, is the average of the Kullback–Leibler divergences D_KL_(*p,a*) and D_KL_(*q,a*):

$$D_{\mathrm{JS}}\left(p,q\right)=\frac{D_{\mathrm{KL}}\left(p,a\right)+D_{\mathrm{KL}}\left(q,a\right)}{2}$$

where *a* is the mean of *p* and *q* and D_KL_(p,q) is the Kullback–Leibler divergence defined as:

$$D_{\mathrm{KL}}\left(p,q\right)=\overset{n}{\underset{i=1}{\sum }}p_{i}\log\left(\frac{p_{i}}{q_{i}}\right),$$

and where p = (p_1_, …. , p_n_) and q = (q_1_, … , q_n_). In essence, the Kullback–Leibler divergence D_KL_(p,q) calculates the mean log fold changes log (p_i_/q_i_). While the Kullback–Leibler divergence measure is widely used, it has one drawback: its value becomes infinite if one of the components of q is zero. In contrast, the Jensen-Shannon divergence always yields a value between 0 and 1. A Jensen-Shannon divergence score of 0 means that two community states are the same. In contrast, a Jensen-Shannon divergence scores of 1 means that the two community states are completely different. The square root of the Jensen-Shannon divergence is called Jensen-Shannon distance.

**Figure 1:**
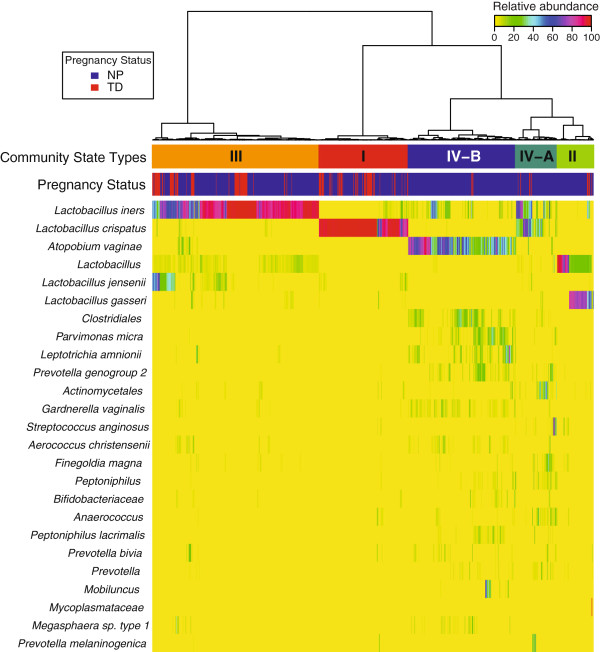
**Heatmap of percentage abundance of microbial taxa found in the vaginal microbial communities of 22 normal pregnant women who delivered at term and 32 non-pregnant women sampled longitudinally.** Ward linkage hierarchical clustering of Jensen-Shannon metric identified five community state types (CST I, II, III, IV-A and IV-B). The upper color bar shows the five community state types while the lower color bar shows the pregnancy status of each sample (NP: Non Pregnant; TD: Term Delivery).

The term “community state type” (CST) is used in microbial ecology to describe a group of community states with similar microbial phylotype composition and abundance [109,112]. Such grouping is desirable in order to reduce dimensionality. Utilizing Jensen-Shannon divergence as a measure of dissimilarity among community states and hierarchical clustering with Ward linkage, five CSTs in the combined dataset of pregnant and non-pregnant women have been identified (Figure 1). Three of the CSTs (CST I, II, III) are dominated by *Lactobacillus* spp. and the remaining two (CST IV-A, IV-B) consist of community states with substantially lower number of *Lactobacillus* spp. than the other CSTs.

### Comparison of community state type frequencies in the non-pregnant state and normal pregnancy

In order to assess significance of differences in frequencies of CSTs between pregnant and non-pregnant women, we considered one CST at a time and created an indicator variable Y, with Y = 1 for samples that belonged to the CST of interest and 0 otherwise. We regressed the CST indicator variable on the pregnancy status using generalized estimation equations (GEE) considering that the response is correlated within patients. The model fitting was performed using the *geepack* package [238] in R (version 2.15), specifying a binomial distribution for the dependent variable (CST indicator), and assuming an exchangeable correlation structure (the response correlation within a subject is similar between all pairs of time points). The odds ratio of belonging to a given CST given that the woman is pregnant was reported together with the significance of the effect (determined by default via a Wald test in *geepack*). *P*-values false discovery rate adjustment for multiple comparisons across the five CSTs was performed and a q-value <0.05 was deemed significant.

### Identification of phylotypes accounting for differences in the structure of vaginal microbiota between the non-pregnant state and normal pregnancy

In order to assess which phylotypes account for the differences in the structure of microbial communities, we modeled relative abundance of one phylotype at a time as a function of pregnancy status, then selected those phylotypes for which there was a significant effect of pregnancy status. Only phylotypes present (based on at least one read count) in 25% or more for the samples were considered in this analysis.

Read count data obtained from a longitudinal experiment design are typically modeled using GEE or linear mixed-effects models by assuming a Poisson or negative binomial distribution of the response. The choice of a Poisson distribution will be justified when the counts variance equals the counts mean, while the negative binomial distribution will be preferred when the mean variance equality cannot be safely assumed.

Several phylotypes were not detected in a large proportion of samples; hence, the frequency of 0 count values in the dataset is larger than expected under a Poisson or negative binomial distribution. For such circumstances, models that can allow for zeroes inflation are more suitable.

In general, the zero-inflated version of a distribution D (for example, negative binomial) of a random variable Y has a probability function of the form:

$$f_{\mathrm{ZID}}\left(y\right)=\pi I\left(y=0\right)+\left(1-\pi \right)f_{D}\left(y\right)$$

where f_D_(y) is the probability function of the distribution D, f_ZID_(y) is the probability function of the zero inflated version of D with an additional parameter *π* as the proportion of additional zeros and I(x) is the indicator function equal to 1 if x is true and equal to 0 otherwise. From the above equation, the probability of y = 0 is equal to *π* + (1 - *π*)f_D_(0), while the probability of y > 0 is (1 - *π*)f_D_(y). Zero inflated models for count data have been used in statistics for at least 20 years [239].

To ensure a proper fit of the count data of each phylotype, we have utilized zero-inflated negative binomial mixed-effects (ZINBLME) models in addition to the simpler negative binomial linear mixed effects (NBLME) and Poisson linear mixed effects (PLME) models. These three types of models were fitted to each phylotype and the model with lowest Akaike Information Criterion (AIC) value was retained. The significance *P*-value for the association between the microbial relative abundance and the group variable was computed only for the best model (smallest AIC).

The mixed effects modeling of the reads count data (dependent variable) on the pregnancy status (independent variable) was performed using the NLMIXED procedure in SAS (version 9.3; SAS, Cary, NC, USA) as discussed elsewhere [240-242]. All three types of models (PLME, NBLME and ZINBLME) had included an offset term (the log of the total number of reads in a given sample) to allow for a comparison in the relative abundance (and not absolute counts) between groups. The random effect in the ZINBLME models was allowed only on the non-zero inflation component (negative binomial mean).

For each of the three types of models, the reported coefficient represents the difference in mean log relative abundance between in samples from pregnant and non-pregnant women that was further converted into a fold change. The *P*-value of the model with the best fit (smallest AIC) was retained and false discovery rate adjustment was applied across the phylotypes. A q-value <0.1 and fold change >1.5 was used to claim significance.

## Results

### Characteristics of the study population

The clinical and demographic characteristics of the pregnant population are displayed in Table 1. The clinical and demographic characteristics of non-pregnant subjects have been previously reported [112]. The present study included 32 non-pregnant women and 22 pregnant women who had a term delivery without complications (gestational age at delivery from 38 to 42 weeks). Non-pregnant women self-sampled with a frequency of twice a week for 16 weeks. The median (interquartile range (IQR)) number of samples with available sequence data was 27.5 samples per participant (IQR: 20.7 to 29). Pregnant women had a median of 6.5 samples per pregnancy (IQR: 6.0 to 7.0).

**Table 1:** Descriptive characteristics of the pregnant woman enrolled in the longitudinal study (n=22)

	**Mean**	**SD**^**a**^	**Minimun**	**Maximum**
Age (years)	24.2	5.2	19	35
Race^b^				
African American	19 (86%)			
White	2 (9%)			
Hispanic	1 (5%)			
Body Mass Index (BMI; kg/m^2^)	29.8	4.9	20.2	39.9
Nulliparity	10 (45%)			
Cesarean delivery	2/22 (9%)			
Gestational age at delivery (weeks)	39.8	1.0	38.1	42.1
Birthweight (grams)	3320	290	2645	4090
Apgar at 1 minute (median)	9.0		7	9
Apgar at 5 minutes (median)	9.0		5	9
Nugent score >=7^c^	2 (9%)			

^a^Standard Deviation.

^b^Non-pregnant women: African American (50%), White (40.6%), Hispanic and others (9.4%).

^c^In at least one sample of a given subject – According to reference 61, Nugent score above 7 correspond to a diagnosis of bacterial vaginosis.

### Characterization of the microbial taxa as a function of depth of coverage

We characterized the vaginal microbiota using pyrosequencing of barcoded 16S RNA genes. The dataset consisted of 2,946,507 high-quality sequences, with an average length of 240 bp. The median number of sequences per sample was 2,878 (IQR: 2,446 to 4,171). Taxonomic assignment of the sequences identified a total of 143 taxa in the vaginal microbiota of the women studied; all 143 taxa were observed both in non-pregnant as well as pregnant women who delivered at term. The taxonomic assignments of vaginal bacterial community members are shown in Additional file 1: Table S1.

### The vaginal microbiota in the non-pregnant state and normal pregnancy

To study the vaginal bacterial communities of pregnant versus non-pregnant women, we hierarchically clustered the vectors of relative abundances of bacterial phylotypes (one per sample) using the Jensen-Shannon divergence metric and Ward linkage [112]. In this study, we refer to a community state as a vector of relative abundances of bacterial phylotypes for a given sample. Community states were clustered into five groups with similar bacterial composition and abundance (Figure 1), referred to as CSTs according to the nomenclature established by Gajer and colleagues [112].

Three of these CSTs were most often dominated by *L. crispatus* (CST I), *L. gasseri* (CST II) and *L. iners* (CST III)*.* Communities that clustered in CST IV-A or IV-B lacked a substantial number of *Lactobacillus* spp. and differed in taxa composition. For example, CST IV-A was characterized by a roughly equal number of *Peptoniphilus*, *Anaerococcus*, *Corynebacterium*, *Finegoldia*, *Prevotella* and a few other taxa. In contrast, those of CST IV-B had higher relative abundance of the genus *Atopobium* and were characterized by the presence of *L. iners* (low relative abundance), *Prevotella*, *Sneathia*, *Gardnerella*, *Ruminococcaceae*, *Parvimonas*, *Mobiluncus* and other taxa previously shown to be associated with bacterial vaginosis [96]. These findings are consistent with previous observations indicating that there is no single “core” microbiota of the human vagina [109]. The relationship between Nugent score and CST was demonstrated. It is noteworthy that CST IV-B was strongly associated with a high Nugent score (defined as 7 to 10) (*P* = 0.013 using a mixed effect model; odds ratio = 24.3).

Table 2 shows the counts of samples assigned to each CST and corresponding percentages stratified by pregnancy status. A dramatic difference in the distribution of frequency of CSTs between non-pregnant and pregnant patients who delivered at term was observed (a decrease of 95% in the odds of observing CST IV-B in pregnant women compared to non-pregnant women).

**Table 2:** Distribution of samples in each community state-type as a function of pregnancy status (non-pregnant vs normal)

**CST/Pregnancy status**	**I**	**II**	**III**	**IV-A**	**IV-B**	**Total**
Non-pregnant women	129 (17%)	68 (8.9%)	268 (35.2%)	79 (10.4%)	217 (28.5%)	761
Normal pregnancy	53 (38.1%)	6 (4.3%)	72 (51.8%)	5 (3.6%)	3 (2.2%)	139

Since Table 2 was generated using correlated samples, standard methods (for example, Fisher tests) cannot be applied to assess significance of differences in frequencies of each CST between pregnant and non-pregnant women. Instead, for each CST (T), a logistic regression GEE model was fitted with the binary response variable (T versus non-T) used as a dependent variable and the pregnancy status used as an independent variable. The coefficients, odds ratios, *P*-values, and q-values for the five GEE models are shown in Table 3. The frequency of CST IV-B (most often dominated by *Atopobium*) was significantly lower in pregnant compared to non-pregnant women. The relative abundance of CST I (dominated by *L. crispatus*) was borderline significantly different between pregnant and non-pregnant women (based on unadjusted *P* = 0.0507 at the 5% significance level).

**Table 3:** Coefficient estimates, odds ratios, p-values and q-values for the association between each community state type with the pregnancy status

**Community state type**^**a**^	**Estimate**^**b**^	**Odds ratio**	**p-value**	**q-value**^**c**^
IV-B	-3.06	0.047	0.00000	0.00001
I	1.09	2.986	0.05076	0.12689
III	0.76	2.136	0.11344	0.18907
IV-A	-1.23	0.292	0.16958	0.21198
II	-0.73	0.482	0.48193	0.48193

^**a**^**Community state type:** a group of community states with similar microbial phylotype composition and abundance identified via unsupervised clustering (Figure 1).

^**b**^**Estimate:** the value of the coefficient in the logistic regression model for a binary variable indicating whether (1) or not (0) a given sample was assigned in the community state named in column 1. The value of the coefficient represents the log of the odds ratio that the sample belongs to the community state indicated in column 1 given that the sample belongs to a pregnant woman (as opposed to a non-pregnant woman).

^**c**^**q-value:** the False Discovery Rate adjusted p-value across all 5 community types that were tested.

### Constancy of the vaginal microbiota in pregnant and non-pregnant women

**Figure 2:**
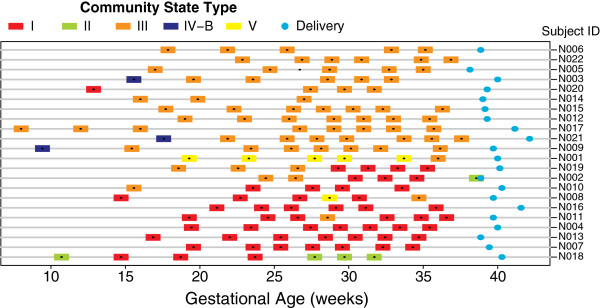
**Profiles of community state types for pregnant women who delivered at term as a function of gestational age.** Gestational age at delivery is indicated by blue solid circles.

Figure 2 shows the profiles of CSTs for pregnant women who delivered at term as a function of gestation time. The CST profiles of pregnant and non-pregnant women are somewhat similar (given smaller number of samples per pregnant woman) except that CST IV-B is rarely present in pregnant women. In particular, none of the pregnant women persist in this CST, which lacks substantial number of *Lactobacillus*, whereas communities of seven non-pregnant women persist in CST IV-B for 16 weeks [112].

**Figure 3:**
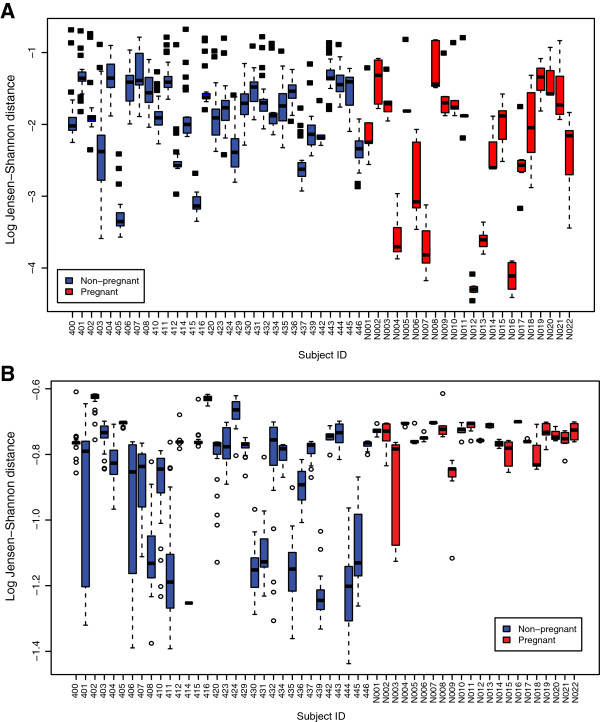
**Estimates of community change over time in non-pregnant and pregnant women who delivered at term. (A)** Jensen-Shannon distances between each community state and the mean community state for each subject. The larger the distance, the higher the instability of the microbial community within a subject. The mean within-subject log Jensen-Shannon distance of pregnant women was significantly lower than that for non-pregnant women (difference in means -0.473 log units; that is, 1.6-fold lower Jensen-Shannon distance, *P* < 0.001). **(B)** Jensen-Shannon distance between each community state and the mean community state of all samples assigned to CST IV-A and CST IV-B. The higher the distance, the less frequently a community enters CST IV-A or CST IV-B. The mean log Jensen-Shannon distance of pregnant women was significantly higher than that for non-pregnant women (difference in means 0.13 log units; that is, 1.14-fold, *P* < 0.001).

Vaginal bacterial communities of most pregnant and non-pregnant women persist in one CST with some intermittent transitions to other CSTs. Is there a difference in constancy of vaginal bacterial communities between pregnant and non-pregnant women? To address this question, we used an approach in which we computed the mean community state within a subject (mean relative abundance of each bacterial phylotype across all samples of a subject), and then the Jensen-Shannon distance was computed between each community state and the mean community state for each subject. These distances are shown in Figure 3A. This is a measure of instability: the larger the distance, the higher the instability of the microbial community within a subject (in other words, community composition changes often over time). To test if the instability was different between pregnant and non-pregnant women, we modeled the log of these Jensen-Shannon distances using a GEE model. The mean within-subject log Jensen-Shannon distance of pregnant women was significantly lower than that for non-pregnant women (difference in means -0.473 log units; that is, 1.6-fold lower Jensen-Shannon distance, *P* < 0.001). This means that vaginal bacterial communities are significantly more stable in pregnant than in non-pregnant women. However, the results indicate that, during pregnancy, the structure of the bacterial community undergoes some change. To characterize the nature of the changes during pregnancy, we evaluated the ability of a community to shift to CST IV (A or B) by computing the Jensen-Shannon distance between each community state and the mean community state of all samples assigned to CST IV-A and CST IV-B (mean relative abundance of each bacterial phylotype across all samples in CST IV-A and CST IV-B). We modeled the log of these Jensen-Shannon distances using a GEE model and found that the mean log Jensen-Shannon distance of pregnant women was significantly higher (further away from CST IV-A or CST IV-B) than that for non-pregnant women (difference in means 0.13 log units; that is, 1.14-fold, *P* < 0.001) (Figure 3B). Altogether, these results indicate that bacterial communities in pregnancy do shift from one CST dominated by *Lactobacillus* spp. to another CST dominated by *Lactobacillus* spp., but rarely to CST IV-A or CST IV-B.

### Identification of phylotypes accounting for differences in the structure of vaginal microbiota between the non-pregnant state and normal pregnancy

Table 3 provides evidence that the vaginal microbiota in women who deliver at term is different from the vaginal microbiota of non-pregnant women. Nonetheless, this analysis does not identify explicitly the phylotypes responsible for differences in the structure of the vaginal microbiota between pregnant and non-pregnant women.

In order to identify phylotypes whose relative abundances were significantly different between pregnant and non-pregnant women, we used statistical models that: 1) were designed for count data modeling (assuming Poisson and negative binomial distributions); and 2) allowed correlated observations from the same individuals (for example, linear mixed effect models); while 3) allowing for extra zeroes in the data since some phylotypes were frequently undetected. Three types of models were fitted for each phylotype, including PLME, NBLME and ZINBLME models. The model type with the smallest AIC value was retained for each phylotype and the *P*-value for group variable (pregnant versus non-pregnant) was computed only for this model. Only phylotypes that were present in at least 25% of all samples were included in the analysis, restricting the number of phylotypes to 28. Table 4 shows the AIC statistics for all three types of models for each phylotype, as well as the estimate, confidence interval and *P*-value for the best (smallest AIC) model. Of interest, out of the 28 phylotypes tested, the relative abundance of 26 was significantly different between the two groups (q-value <0.1 and fold change >1.5). Four of the significant phylotypes (*L. vaginalis*, *L. crispatus*, *L. gasseri* and *L. jensenii*) were more abundant in pregnant than non-pregnant women (Additional file 2: Figures S1 show box plots of the relative abundances of all significant phylotypes listed in Table 4). The NBLME model provided the optimal fit for a majority of phylotypes, indicating that there is over-dispersion in the sequence count data and, hence, the Poisson distribution may be too restrictive for the observed count data. This finding is in agreement with previous observations [243]. About a quarter of the significant phylotypes showed zero inflation; therefore, the zero-inflation version of the negative binomial model (ZINBLME) provided the optimum fit based on AIC values. The SAS code and input dataset used to generate the results presented in Table 4 are provided in Additional files 3 and 4, respectively.

**Table 4:** Differential relative abundance of microbial phylotypes between pregnant and non-pregnant women and statistics for the phylotype level analysis

**Phylotypes**		**PLME AIC**^**a,d**^	**NBLME AIC**^**b,d**^	**ZINBLME AIC**^**c,d**^	**Best AIC**^**d**^	**Estimate**	**Lower 95% CI**	**Upper 95% CI**	**Fold change**	**p-value**	**q-value**^**e**^
**Phylotypes less abundant in pregnancy**											
	*Clostridiales Family XI Incertae Sedis*	3488.3	2996.9	2998.9	NBLME	-7.095	-9.020	-5.169	-1205.6	0.0001	0.0002
	*Anaerococcus vaginalis*	3043.8	2748.2	2759.9	NBLME	-5.873	-7.260	-4.486	-355.4	0.0001	0.0002
	*Anaerococcus*	5252.6	3804.3	3806.3	NBLME	-5.557	-6.605	-4.509	-259.0	0.0001	0.0002
	*Prevotella genogroup 2*	5510.3	4498.2	4504.2	NBLME	-5.019	-5.655	-4.384	-151.3	0.0001	0.0002
	*Peptoniphilus*	4707.8	4431.3	4433.3	NBLME	-4.921	-5.670	-4.171	-137.1	0.0001	0.0002
	*Streptococcus anginosus*	3285.4	2565.8	2583.8	NBLME	-4.629	-5.748	-3.511	-102.4	0.0001	0.0002
	*Actinomycetales*	5672.8	5110.1	5112.1	NBLME	-4.546	-5.281	-3.811	-94.2	0.0001	0.0002
	*Leptotrichia amnionii*	7299.3	3889.1	3893.3	NBLME	-4.490	-5.359	-3.621	-89.1	0.0001	0.0002
	*Finegoldia magna*	4687.1	4300.6	4302.6	NBLME	-4.174	-4.958	-3.391	-65.0	0.0001	0.0002
	*Prevotella*	4540	4094.7	4096.7	NBLME	-3.870	-4.502	-3.238	-48.0	0.0001	0.0002
	*Clostridiales*	5864.5	4852.6	NA	NBLME	-3.373	-4.274	-2.472	-29.2	0.0001	0.0002
	*Atopobium*	3853.1	3275	3261.6	ZINBLME	-3.268	-3.943	-2.593	-26.3	0.0001	0.0002
	*Bacteria*	3167.5	3033	3040.1	NBLME	-3.083	-3.921	-2.245	-21.8	0.0001	0.0002
	*Prevotella.bivia*	4178.9	3043	3045	NBLME	-3.038	-4.089	-1.986	-20.9	0.0001	0.0002
	*Eggerthella*	3149.7	3083.7	3065.2	ZINBLME	-1.936	-2.813	-1.060	-6.9	0.0001	0.0002
	*Gardnerella vaginalis*	5472	5105.1	5076.7	ZINBLME	-1.760	-2.253	-1.266	-5.8	0.0001	0.0002
	*Dialister*	4048.5	3939.6	3940.9	NBLME	-1.399	-2.147	-0.651	-4.1	0.0003	0.0004
	*Ureaplasma*	2819	2700.6	2707.8	NBLME	-1.153	-1.817	-0.490	-3.2	0.0007	0.0010
	*Lactobacillus*	10572	9170.2	9172.2	NBLME	-0.726	-1.169	-0.283	-2.1	0.0013	0.0017
	*Atopobium vaginae*	12734	6971.9	7024.5	PLME	-2.381	-3.946	-0.816	-10.8	0.0029	0.0037
	*Parvimonas micra*	4512.2	3835.1	3821.7	ZINBLME	-4.202	-7.609	-0.795	-66.8	0.0157	0.0183
	*Bifidobacteriaceae*	4056.8	3989.9	3991.9	NBLME	-0.660	-1.429	0.110	-1.9	0.0927	0.0998
**Phylotypes more abundant in pregnancy**											
	*Lactobacillus vaginalis*	2489.2	2467.9	2458.2	ZINBLME	1.704	1.190	2.218	5.5	0.0001	0.0002
	*Lactobacillus jensenii*	6544.1	5564.1	5549.7	ZINBLME	1.549	1.453	1.645	4.7	0.0001	0.0002
	*Lactobacillus crispatus*	11702	8094	8263.2	NBLME	0.754	0.212	1.295	2.1	0.0064	0.0078
	*Lactobacillus gasseri*	6917.3	4412.4	NA	NBLME	1.193	0.214	2.172	3.3	0.0170	0.0190
**Non-significantly different phylotypes**											
	*Lactobacillus iners*	18755	12576	12604	NBLME	0.165	-0.136	0.466	1.2	0.2824	0.2929
	*Aerococcus christensenii*	4321.2	3986.9	3957.1	ZINBLME	-0.425	-1.251	0.401	-1.5	0.3132	0.3132

^a^PLME: Poisson Linear Mixed Effects Model.

^b^NBLME: Negative Binomial Linear Mixed Effects.

^c^ZINBLME: Zero-Inflated Negative Binomial Mixed-Effects Model.

^d^AIC: Akaike Information Criterion.

^e^q-value is p-value after adjustment for false-discovery rate (0.1).

Some of the selected phylotypes are defined at the genus and some at the species level (for example, *Anaerococcus* and *Anaerococcus vaginalis*), respectively. A genus level phylotype corresponds to a set of sequences that could not be reliably identified at the species level for any known species of the given genus. Thus, in the case of *Anaerococcus* and *Anaerococcus vaginalis*, the first phylotype corresponds to reads that cannot be taxonomically assigned to any known species of *Anaerococcus* and might represent uncharacterized species of *Anaerococcus*, whereas the phylotype *Anaerococcus vaginalis* consists of reads that are classified as corresponding to that species.

## Discussion

### Principal findings of the study

Using sequence-based methods (rather than cultivation techniques) to characterize the vaginal microbiota in a longitudinal study of normal pregnant women and non-pregnant women, we established that: 1) at the bacterial community level, CST IV-B (characterized by high relative abundance of species of *Atopobium* as well as the presence of *Prevotella*, *Sneathia*, *Gardnerella*, *Ruminococcaceae*, *Parvimonas*, *Mobiluncus* and other taxa previously shown to be associated with bacterial vaginosis) was rarely observed in pregnant women who delivered at term; 2) the vaginal microbiota of normal pregnant women who deliver at term was different from that of non-pregnant women (higher abundance of *L. vaginalis*, *L. crispatus*, *L. gasseri* and *L. jensenii* and lower abundance of 22 other phylotypes in normal pregnancy); 3) the stability of the vaginal microbiota of pregnant women was higher than that of non-pregnant women; and 4) during normal pregnancy, bacterial communities do shift from one CST dominated by *Lactobacillus* spp. to another CST dominated by *Lactobacillus* spp. but rarely to CST IV-A or CST IV-B.

### The vaginal microbiota of normal pregnant women

This is the first longitudinal study of the vaginal microbiota in normal pregnancy where samples have been frequently collected and microbial composition has been characterized using high-throughput pyrosequencing of the 16S rRNA gene. Previous studies have used a cross-sectional approach [213] and sparse sampling [212]. Some have used low resolution microbiological and molecular techniques [211,212] to characterize the microbial communities. The methodology used in the present study provides a less biased, in-depth characterization of the bacterial composition and abundance of the vaginal microbiota. The major finding of this study is that normal pregnant women maintain (throughout the entire pregnancy) vaginal CSTs dominated by *Lactobacillus* spp*.* This is in contrast with the observations made in the non-pregnant state, in which there were fluctuations between CSTs lacking a substantial number of *Lactobacillus* spp*.* and those that are dominated by members of this genus [112]*.*

In a previous study, we focused on non-pregnant women and characterized five different CSTs (CST I to V); CST I, II, III and V were characterized by a predominance of *Lactobacillus* spp. CST IV was characterized by a low abundance of *Lactobacillus* spp. and a predominance of other phylotypes, mainly of anaerobic bacteria. This CST was further subdivided into IV-A and IV-B based on hierarchical clustering [112]. The major difference between the two is that CST IV-B has a higher abundance of *Atopobium*, while CST IV-A has a more even microbial composition including the following phylotypes: *Peptoniphilus*, *Anaerococcus*, *Corynebacterium*, *Finegoldia* and *Prevotella*. We have also reported that CST IV-A and CST IV-B were more common in certain ethnic groups (African-American and Hispanic) and were associated with a higher vaginal pH and high Nugent score [109]. In the current study focusing on pregnant women, we identified five of the six CSTs previously described: I, II, III, IV-A and IV-B. We did not find CST V. The most likely explanation for this is that the majority of women enrolled in the present study were African-American, and CST V was previously observed in only 1% of such women [109]. Given the sample size of the current study (n = 22 pregnant women) and the ethnic composition (90% African-American), the lack of representation of CST V is not unexpected. Therefore, these findings do not mean that other studies of the microbiota of pregnant women using a different population would not identify CST V.

### Stability of the vaginal microbiota during pregnancy

During normal pregnancy, bacterial communities are more stable than in the non-pregnant state; however, some changes do occur. For example, bacterial communities commonly transitioned from one *Lactobacillus*-dominated CST to another, but rarely to CST IV-A or CST IV-B. This is a reflection of the importance of *Lactobacillus* spp. in the vaginal ecosystem during pregnancy. Such an interesting feature can be interpreted to represent an adaptation of the microbial community and the host to maximize reproductive fitness. We propose that the enhanced stability confers greater resilience and has a protective role against ascending infection of the genital tract, which is risk factor for preterm delivery [244-246] and other conditions such as a sonographic short cervix [247-249], cervical insufficiency [250-254], preterm labor in twin gestations [255-257], vaginal bleeding in the third trimester [258], placenta previa [259,260], or some cases of fetal death [261-265]. The mechanisms by which bacterial community stability promotes health in the vaginal niche remain to be determined.

### Is the vaginal microbiota unique during pregnancy?

Our findings indicate that there are phylotypes with relative abundance that differ between pregnant and non-pregnant women. Specifically, four phylotypes (*L. vaginalis*, *L. jensenii*, *L. crispatus* and *L. gasseri*) had higher relative abundance in pregnant than in non-pregnant women. We identified another 22 phylotypes that had lower relative abundance in pregnant than non-pregnant women (Table 4); many of these phylotypes are associated with CST IV-A and CST IV-B. Interestingly, the relative abundance of *L. iners* was not significantly different between the two groups. This finding might reflect a lack of optimal protection by this common *Lactobacillus* sp. [109] and deserves further investigation. Aagaard and colleagues [213] have proposed that there is a microbiota signature of pregnancy based upon a cross-sectional study of pregnant (n = 24) and non-pregnant women (n = 60). Using a random forest algorithm, pregnancy was well predicted by relative abundances of different phylotypes in vaginal fluid. At this point, even though there are differences in microbial compositions between the pregnant and non-pregnant state, there is no evidence that these differences are specific to pregnant women. Further, it is unclear if a microbial signature of pregnancy could have utility for diagnostic purposes.

It is possible that the composition of the vaginal microbiome associated with pregnancy may have functional (that is, metabolic, immune) implications for the host [266]. An alternative interpretation is that changes in the microbiota are a consequence of the physiological state of pregnancy. During the course of the menstrual cycle, stability of microbial communities is higher at the time when estrogen concentrations are high (14 and 21 days) [112]. This has been attributed to the effect of estrogens on the maturation of the vaginal epithelium, resulting in the accumulation of glycogen on the upper layer of the epithelium [267-270]. Glycogen is a carbon source metabolized to lactic acid by *Lactobacillus* spp., causing a low vaginal pH [24,26,29]. Further research is required to determine if the relationship between high estrogens and increased stability is causal.

### Strengths and limitations

The major strengths of this work are: 1) the longitudinal nature of the study, which allows characterization of the vaginal microbiota over time; 2) the frequent sampling protocol - this allowed characterization of the dynamics of the bacterial communities in pregnancy to an extent not done before; 3) the quality of the sequence-based techniques (16S rRNA) which reduced bias over other methods, including cultivation techniques; 4) the analytical methods that took into consideration changes over time on the same subject, therefore increasing the power of detection of differences between clinical groups; and 5) inclusion of relevant clinical groups: non-pregnant and normal pregnant women. These strengths allowed meaningful differences to be found among these clinical groups. The use of primer 27 F could be a limitation of this study; this primer may have underestimated the true relative abundance of 16S rRNA genes of Bifidobacteriaceae in general, and those of the genus *G. vaginalis*, a bacterium commonly found in the vagina of women who experience bacterial vaginosis. The selection of optimal PCR primers is a subject of considerable ongoing discussion in the field of microbiome studies. Unfortunately, there is no consensus, nor a perfect set of primers. In this study, we followed the recommendations of the NIH-funded Human Microbiome Project (http://www.hmpdacc.org/). Another potential limitation of the study is the sample size, which included 22 pregnant women who delivered at term. Yet, despite the apparently limited sample size, the identification of significant differences provides evidence that the study of the vaginal microbiota during pregnancy can yield important insights into the relationship between the structure and dynamics of microbial communities and pregnancy outcome. Further studies are required to confirm these findings, extend the observations and elucidate the role of microorganisms in adverse pregnancy outcome.

## Conclusion

This is the first longitudinal study of the human vaginal microbiota in pregnancy. We demonstrate differences in the vaginal bacterial community structure between normal pregnant and non-pregnant women and show that pregnancy is characterized by a greater degree of stability than observed in non-pregnant women. We established the baseline stability patterns of the vaginal microbiota in pregnancy. This could serve as the basis to study the relationship between the vaginal microbiota and adverse pregnancy outcomes. The characterization of the vaginal microbiota in pregnancy has the potential to yield information of prognostic, diagnostic and therapeutic value.

## Abbreviations

AIC: Akaike Information Criterion; bp: base pair; CST: community state type; GEE: generalized estimation equation; IQR: interquartile range; NBLME: negative binomial linear mixed effect; NIH: National Institutes of Health; PCR: polymerase chain reaction; PLME: Poisson linear mixed effect; ZINBLME: zero-inflated negative binomial mixed-effect.

## Competing interests

The authors declare that they have no competing interests.

## Authors’ contributions

RR, SSH, PG, AT and JR conceived the study. RR, SSH, LN, MG, RFL, PC, JM and TC performed the clinical sampling and samples management. DWF performed DNA extraction, 16S rRNA gene amplification and sequencing. DWF, PG and JR processed the sequence data. PG and AT performed the statistical analyses. RR, SSH, PG, AT and JR wrote the manuscript. All authors read and approved the final manuscript.

## Supplementary Material

Additional file 1: Table S1 Taxonomic assignments, total 16S rRNA gene sequences assigned to each taxa, and metadata. Click here for file

Additional file 2: Figure S1 Box plots of relative abundances of all phylotypes that have statistically significantly different relative abundance between pregnant and non-pregnant women. Click here for file

Additional file 3 **SAS code used to generate the results presented in Table **4**.** Click here for file

Additional file 4 **Input dataset for SAS code used to generate the results presented in Table **4**.** Click here for file
