# Correction: The composition and stability of the vaginal microbiota of normal pregnant women is different from that of non-pregnant women

**DOI:** 10.1186/2049-2618-2-10

**Published:** 2014-04-15

**Authors:** Roberto Romero, Sonia S Hassan, Pawel Gajer, Adi L Tarca, Douglas W Fadrosh, Lorraine Nikita, Marisa Galuppi, Ronald F Lamont, Piya Chaemsaithong, Jezid Miranda, Tinnakorn Chaiworapongsa, Jacques Ravel

**Affiliations:** 1 Perinatology Research Branch, Program for Perinatal Research and Obstetrics, Division of Intramural Research, Eunice Kennedy Shriver National Institute of Child Health and Human Development, NIH, Bethesda, MD and, Detroit, MI, USA; 2 Department of Obstetrics and Gynecology, University of Michigan, Ann Arbor, MI, USA; 3 Department of Epidemiology and Biostatistics, Michigan State University, East Lansing, MI, USA; 4 Department of Obstetrics and Gynecology, Wayne State University School of Medicine, Detroit, MI, USA; 5 Institute for Genome Sciences, University of Maryland School of Medicine, Baltimore, MD, USA; 6 Department of Microbiology and Immunology, University of Maryland School of Medicine, Baltimore, MD, USA; 7 Department of Obstetrics and Gynaecology, University of Southern Denmark, Odense, Denmark; 8 Division of Surgery, University College, Northwick Park Institute for Medical Research Campus, London, UK

## Correction

We have identified an error in Additional file 1: Table S1 in this article [1], whereby the ethnicities were inverted for some of the subjects. An amended Additional file 1: Table S1 is published here, with the corrected ethnicities highlighted.

## Supplementary Material

Additional file 1: Table S1 Taxonomic assignments, total 16S rRNA gene sequences per taxa, and metadata. Click here for file
