# Supplementary figures and images for: The composition and stability of the vaginal microbiota of normal pregnant women is different from that of non-pregnant women
Source: Microbiome. 2014 Feb 3;2:4. doi: 10.1186/2049-2618-2-4 (PMC3916806; doi:10.1186/2049-2618-2-4)

Figure S1

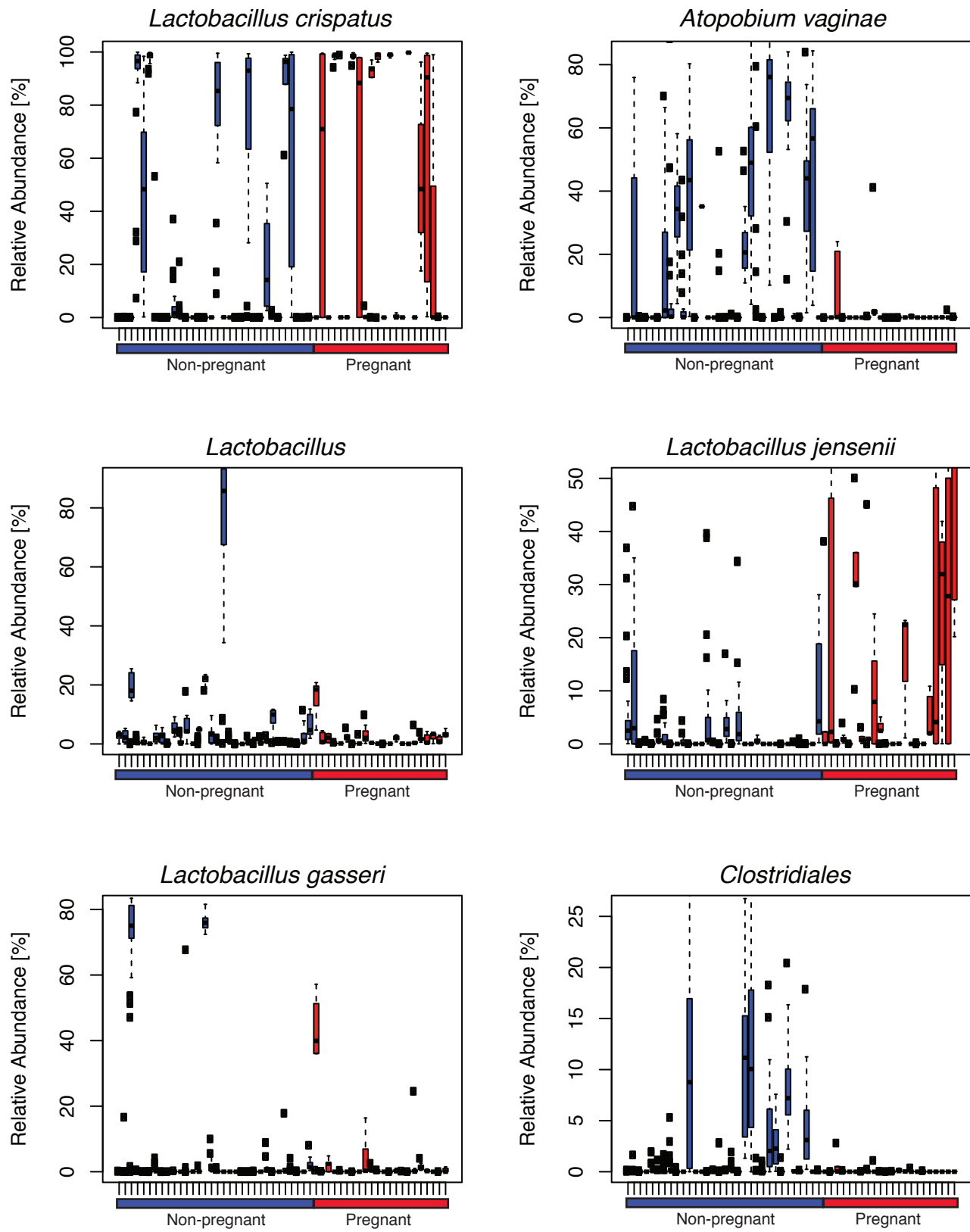

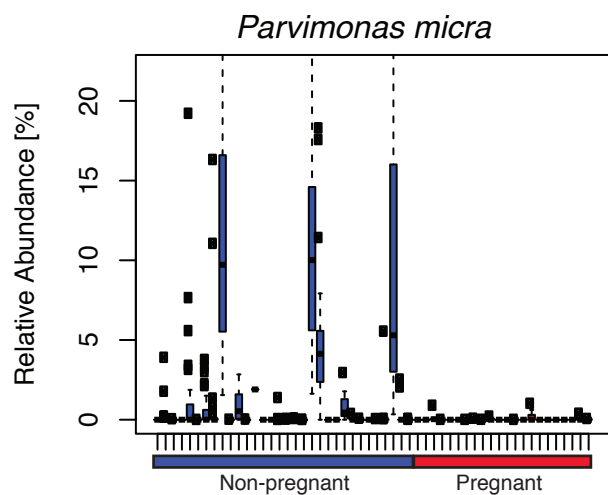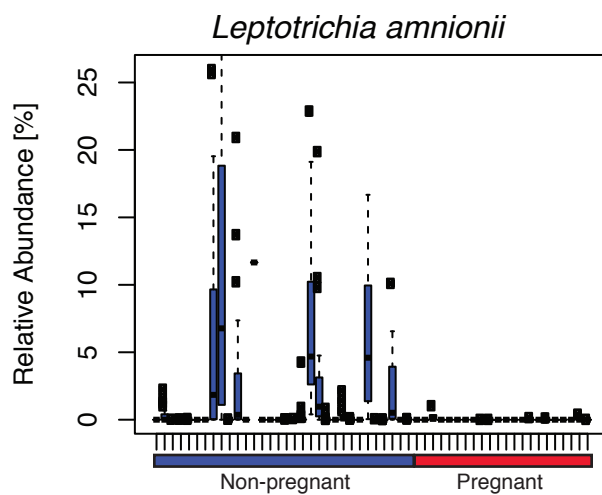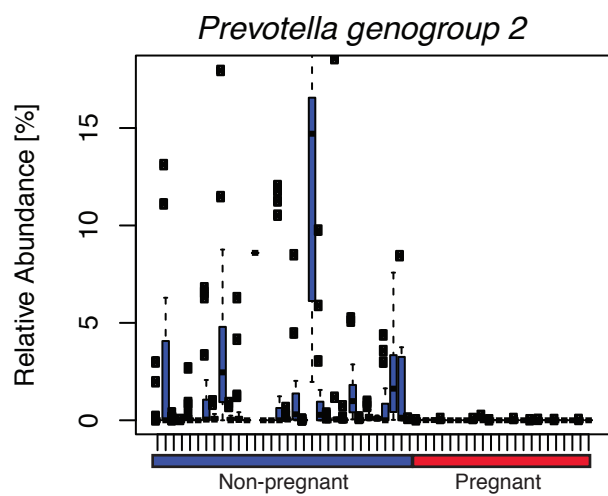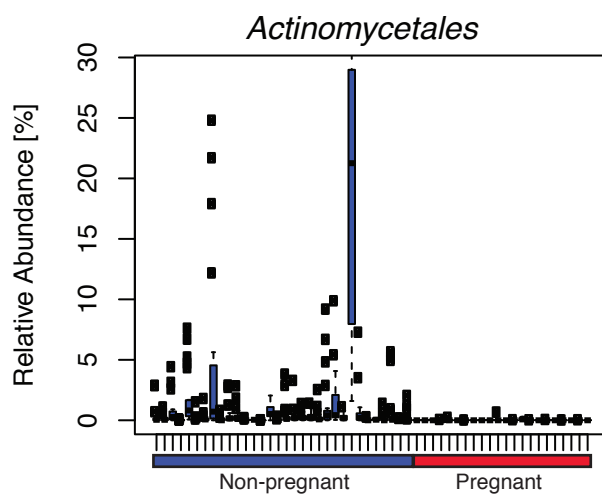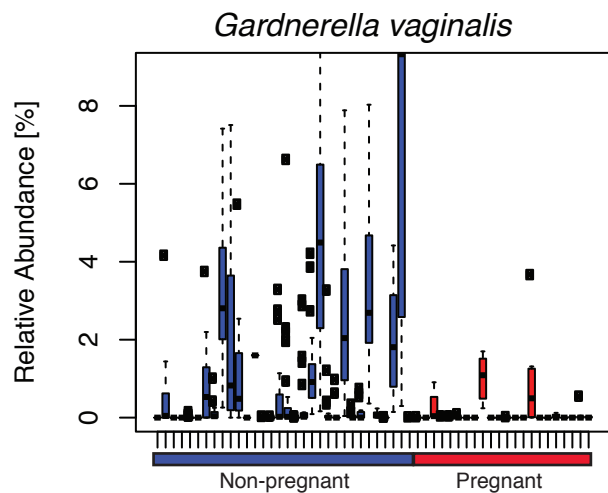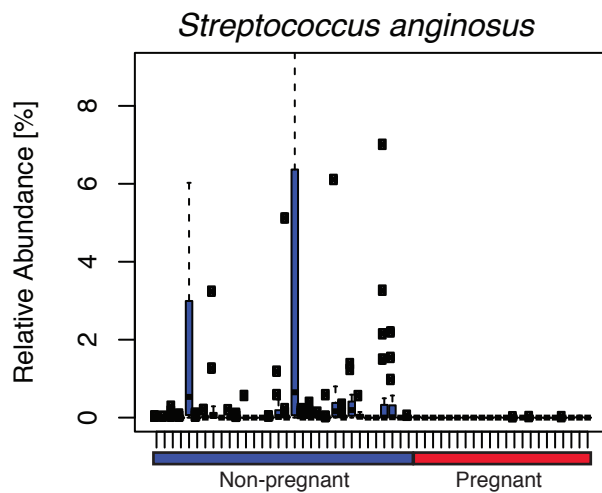

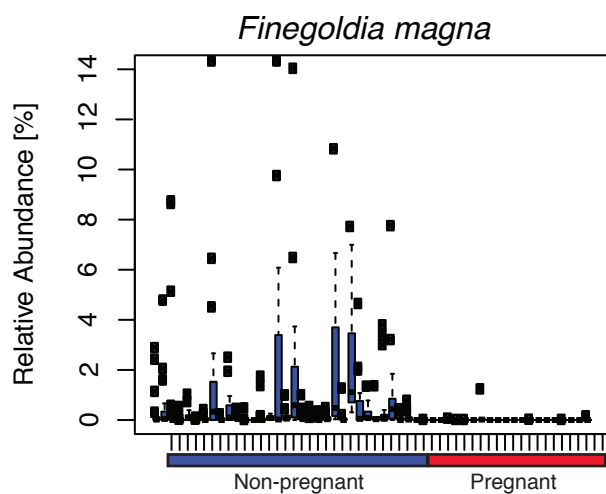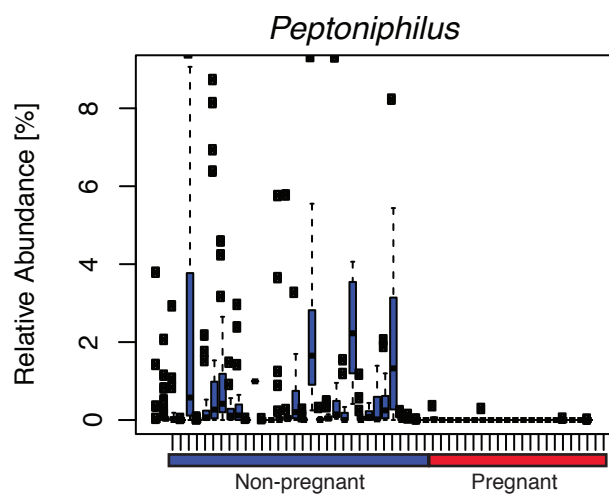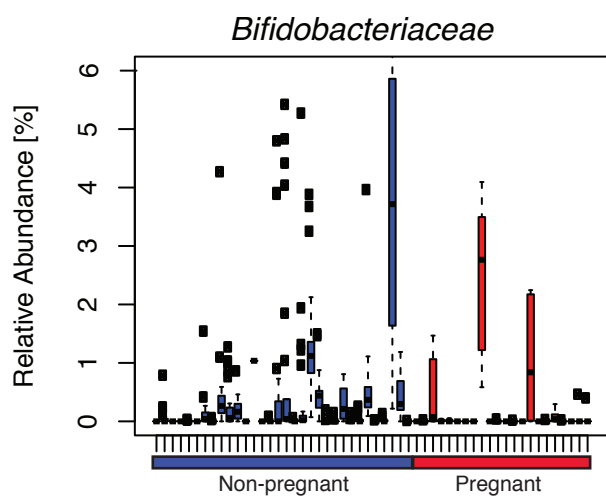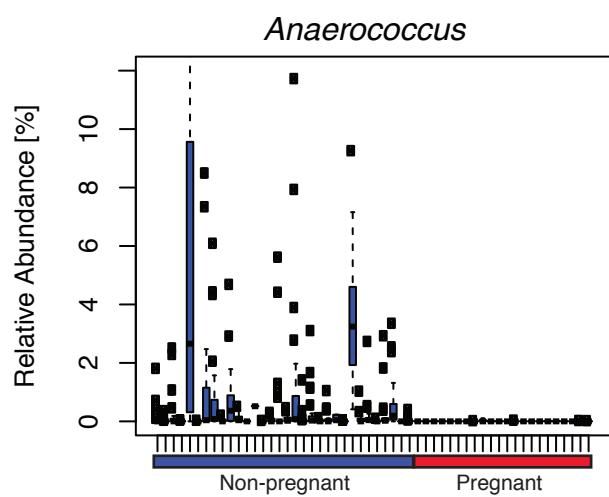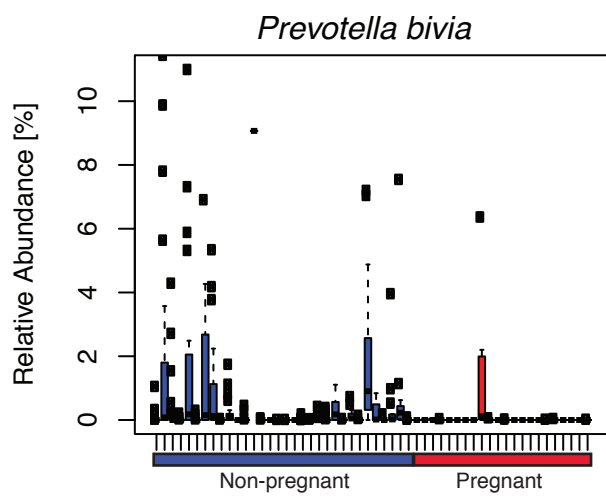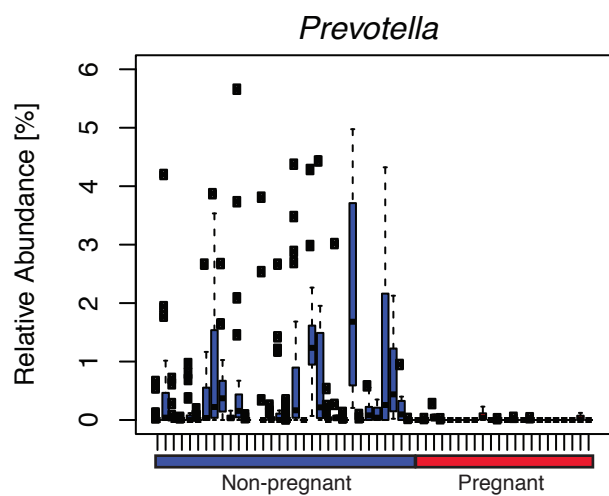

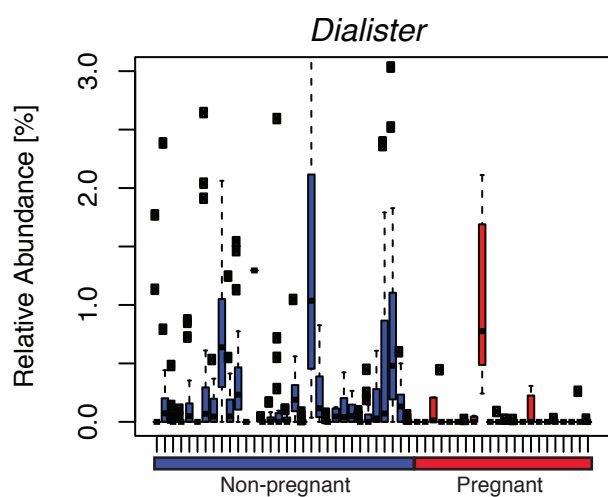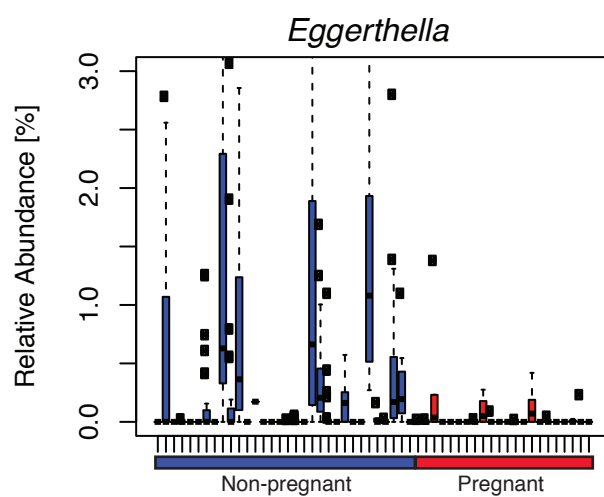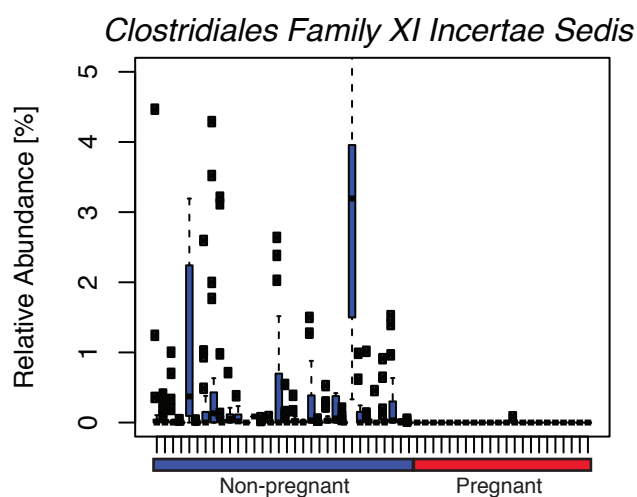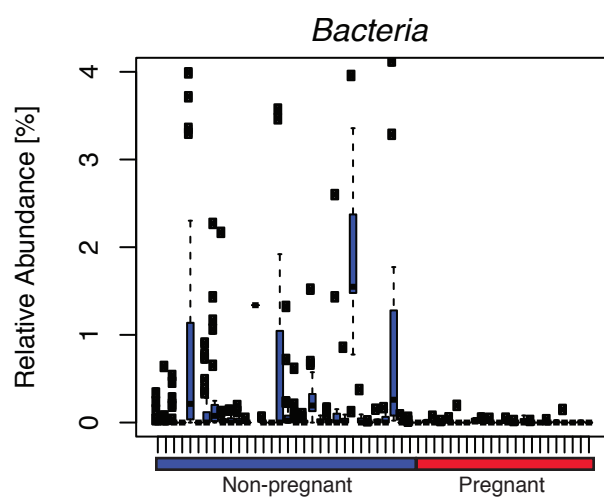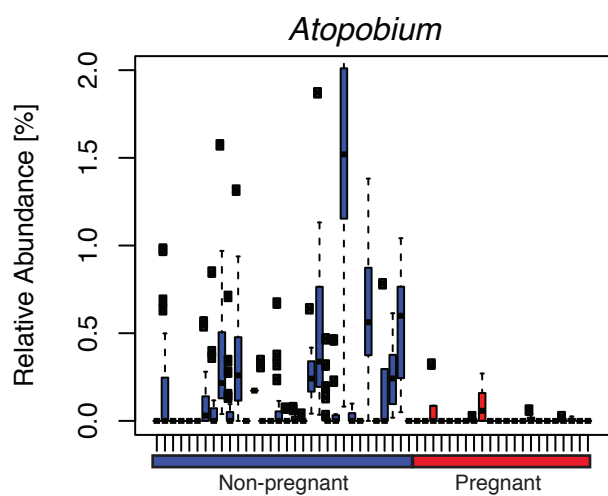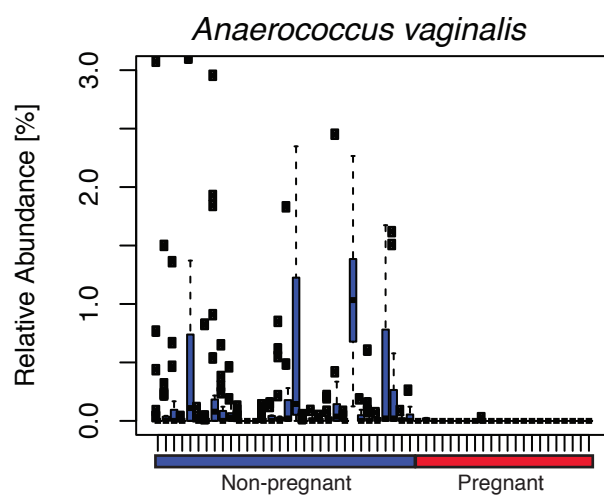

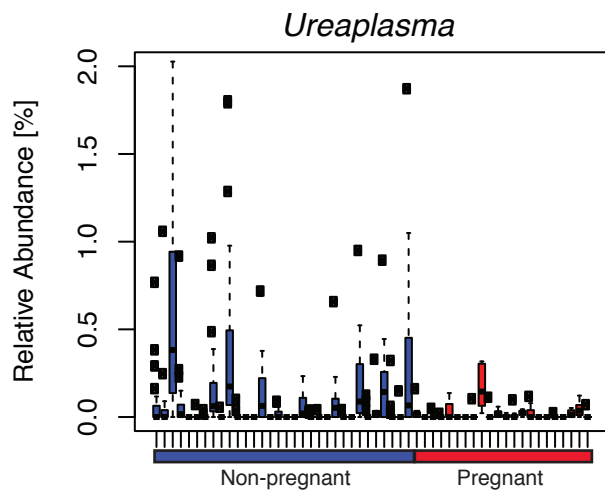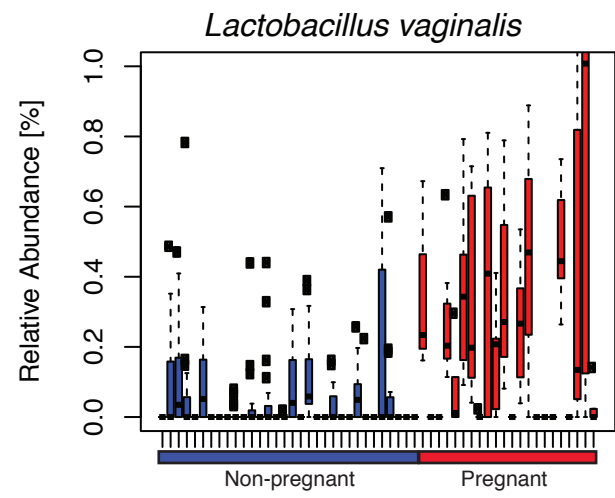

Supplement: Additional file 2: Figure S1 — Box plots of relative abundances of all phylotypes that have statistically significantly different relative abundance between pregnant and non-pregnant women. [file 2049-2618-2-4-S2.pdf]
