# Supplementary material for: The composition and stability of the vaginal microbiota of normal pregnant women is different from that of non-pregnant women
Source: Microbiome. 2014 Feb 3;2:4. doi: 10.1186/2049-2618-2-4 (PMC3916806; doi:10.1186/2049-2618-2-4)
Supplement: Additional file 3 — SAS code used to generate the results presented in Table 4. [file 2049-2618-2-4-S3.doc]

**Additional File 1: SAS code used to generate the results in Table 5**

This appendix provides the SAS code that we used to fit the three types of models that we have employed for testing relative abundance differences between pregnant and non-pregnant women for each OTU. The code below was run using SAS version 9.3, and requires as input the sequence counts data as formatted in the file *allTD_long.csv*. The first three rows of this table are given below, where *ID* represents the identifier of a given subject; *nReads* is the total number of reads in the sample, *DX* is the group indicator (0=non-pregnant; 1=pregnant), *Y* is the number of sequences (count), *Spec* gives the name of the OTU, and *Ind* is a numeric indicator for the OTU (1,…,28).

| **ID** | **nReads** | **DX** | **Y** | **Spec** | **Ind** |
| --- | --- | --- | --- | --- | --- |
| 432 | 2209 | 0 | 4 | Lactobacillus.iners | 1 |
| 424 | 4485 | 0 | 7 | Lactobacillus.iners | 1 |
| 439 | 2447 | 0 | 175 | Lactobacillus.iners | 1 |

The code below produces two files for each of the three types of models: one file with the coefficients and p-values from models (file name postfix C), and one file with the models fit quality including AIC values (file name postfix F).

As a first example, for the first OTU in file allTD_long.csv (*Ind*=1, *Spec*=Lactobacillus.iners), the model with the smallest AIC was the NBLME. Its AIC value shown in Table 5 is 12576. This AIC value can be found in the output of the sas code in the table NBLME_F.csv that gives the model fit statistics. The coefficient for this model representing the log fold change in relative abundance between pregnant and non-pregnant women (see column *Estimate* in Table 5), is 0.1649, value that can be found in the output table generated by the sas code named NBLME_C.csv (column *Estimate* for *Ind*=1 and *Parameter* b1). The corresponding p-value is given by the column *Probt*=0.28.

As second example, for the 25^th^ OTU in file allTD_long.csv (*Ind*=25, *Spec* =Atopobium), the model with the smallest AIC was the zero inflated negative binomial: ZINBLME (see Table 5). Its AIC value shown in Table 5 is 3261.6. This AIC value can be found in the output of the sas code in the table ZINBLME_F.csv that gives the model fit statistics. The coefficient for this model representing the log fold change in relative abundance between pregnant and non-pregnant women (see column *Estimate* in Table 5), is -3.267, value that can be found in the output table generated by the sas code named NBLME_C.csv (column *Estimate* for *Ind*=25 and Parameter b1). The corresponding p-value is given by the column *Probt*<.0001.

**SAS code:**

**proc** **import** datafile='C:\allTD_long.csv'

out =mega

dbms=csv

replace;

getnames=yes;

**RUN**;

**data** newmega;

set mega;

myof=log(nReads);

if DX=**0** then x = **0** ;else x=**1**;

**run**;

/* PLME: Linear Mixed-effects Poisson*/

**proc** **nlmixed** data=newmega; by Ind;

parameters b0=**0** b1=**0** s2u=**1**;

lambda = exp(b0+b1*x+myof+u);

model Y~ poisson(lambda);

ODS OUTPUT ParameterEstimates = LMEPC;

ODS OUTPUT FitStatistics=LMEPF;

random u~normal(**0**,s2u) subject =ID;

**run**;

/* NBLME: Linear Mixed-effects Negative Binomial*/

**proc** **nlmixed** data=newmega; by Ind;

parameters b0=**0** b1=**0** s2u=**1** k=**1**;

linp = b0 + b1*x+myof+u;

mu = exp(linp);

p = **1**/(**1**+mu*k);

model y ~ negbin(**1**/k,p);

ODS OUTPUT ParameterEstimates = LMENBC;

ODS OUTPUT FitStatistics=LMENBF;

random u~normal(**0**,s2u) subject =ID;

**run**;

/* NBZILME: Linear Mixed-effects Negative Binomial with Zero Inflation*/

**proc** **nlmixed** data=newmega; by Ind;

parameters b0=**0** b1=**0** s2u=**1** a0=**0** k=**1**;

eta_zip = a0;

p0_zip = **1** / (**1** + exp(eta_zip));

eta_nb = b0 + b1*x+myof+u;

mean = exp(eta_nb);

p0 = p0_zip +

(**1**-p0_zip)*exp(-(Y+(**1**/k))*log(**1**+k*mean));

p_else = (**1**-p0_zip)*

exp(lgamma(Y+(**1**/k)) - lgamma(Y+**1**) - lgamma(**1**/k) +

Y*log(k*mean) - (Y+(**1**/k))*log(**1**+k*mean));

if Y=**0** then loglike = log(p0);

else loglike = log(p_else);

model Y ~ general(loglike);

ODS OUTPUT ParameterEstimates = LMENBZIC;

ODS OUTPUT FitStatistics=LMENBZIF;

random u~normal(**0**,s2u) subject =ID;

**run**;

/* For each of the 3 types of models create 2 files*/

/* One file with the coefficients and p-values from models; postfix "C"*/

/* One file with the models fit quality including AIC values; postfix "F"*/

**proc** **export** data=LMEPC

outfile= C:\PLME_C.csv'

dbms=csv

replace;

**run**;

**proc** **export** data=LMEPF

outfile='C:\PLME_F.csv'

dbms=csv

replace;

**run**;

**proc** **export** data=LMENBC

outfile='C:\NBLME_C.csv'

dbms=csv

replace;

**run**;

**proc** **export** data=LMENBF

outfile='C:\NBLME_F.csv'

dbms=csv

replace;

**run**;

**proc** **export** data=LMENBZIC

outfile='C:\NBZILME_C.csv'

dbms=csv

replace;

**run**;

**proc** **export** data=LMENBZIF

outfile='C:\NBZILME_F.csv'

dbms=csv

replace;

**run**;
